# Validation of the World Health Organization Disability Assessment Schedule in people with severe mental disorders in rural Ethiopia

**DOI:** 10.1186/s12955-017-0647-3

**Published:** 2017-04-05

**Authors:** Kassahun Habtamu, Atalay Alem, Girmay Medhin, Abebaw Fekadu, Michael Dewey, Martin Prince, Charlotte Hanlon

**Affiliations:** 1 Department of Psychiatry, School of Medicine, College of Health Sciences, Addis Ababa University, Addis Ababa, Ethiopia; 2 School of Psychology, College of Education and Behavioral Studies, Addis Ababa University, P.O.BOX: 1176, Addis Ababa, Ethiopia; 3 Aklilu Lemma Institute of Pathobiology, Addis Ababa University, Addis Ababa, Ethiopia; 4 King’s College London, Institute of Psychiatry, Psychology and Neuroscience, Centre for Affective Disorders, London, UK; 5 Centre for Global Mental Health, Institute of Psychiatry, Psychology and Neuroscience, Kings College London, London, UK

**Keywords:** Disability, Confirmatory factor analysis, Mental disorders, Validation, Africa, Ethiopia, Psychometric properties, Sensitivity to change

## Abstract

**Background:**

The World Health Organization Disability Assessment Schedule (WHODAS-2.0) has been adapted and validated in several cultures, but data on performance in the African context are lacking. The aim of the study was to evaluate the validity and psychometric properties of the WHODAS-2.0 among people with severe mental disorders (SMD) and their caregivers in a rural African setting.

**Methods:**

The content validity of the 36 item WHODAS was assessed using free listing and pile sorting in 36 community members. Cognitive interviewing was conducted with 20 people with SMD and 20 caregivers to assess comprehensibility. Convergent validity and sensitivity to change were evaluated in a facility-based cohort study of new or acutely relapsed cases of people with SMD (*n* = 150) and their caregivers (*n* = 150) consecutively recruited from a psychiatric clinic. A repeat assessment was conducted in a sub-sample (*n* = 84) after 6 weeks. Confirmatory factor analysis was used to evaluate construct validity in people with SMD (*n* = 250) and their caregivers (*n* = 250).

**Results:**

Internal consistency of the items of the overall scale and each domain ranged from very good (alpha = 0.82) to excellent (alpha = 0.98). Scores on the WHODAS-2.0 correlated highly with a locally developed measure of functioning (*r* = 0.88) and moderately with clinical symptom severity (*r* = 0.52). The WHODAS- 2.0 was sensitive to treatment changes (effect size = 0.50). As hypothesized, the six sub-scales loaded highly onto the general disability factor and each item loaded significantly onto their respective domains. The factor loadings of each item in the one factor model of the brief version of WHODAS (12 item) were also high. For both 12- and 36-item scales the goodness of fit indices, were close to, but outside of, recommended ranges. The caregiver data of both the 36 and 12 item versions had similar psychometric properties, but higher mean values and better responsiveness to change.

**Conclusions:**

Our study showed that both the 12 and 36 item versions of the WHODAS 2.0 have acceptable validity and psychometric properties and can be used as a cross-cultural measure; however, careful and rigorous adaptation is required for rural African settings.

**Electronic supplementary material:**

The online version of this article (doi:10.1186/s12955-017-0647-3) contains supplementary material, which is available to authorized users.

## Background

Cross-cultural measurement of functional impairment resulting from health conditions is important for estimating the global burden of disease and for comparing disease burden across settings [1] and across different types of health condition [2]. Standardised instruments exist which are purported to measure day-to-day functioning across health conditions and sociocultural settings [3]. The validity of this universalistic or ‘etic’ approach has been questioned, as most cross-cultural measures have been developed in Western, high-income country settings and focus on activities or tasks that may not include those that are relevant in low- and middle-income countries (LAMICs) [4]. An alternative approach is to develop locally relevant measures of functioning which are sensitive to gender and may have greater cultural validity [4]. The limitation with this more contextually grounded approach is a loss of generalizability which makes comparison across settings problematic.

The World Health Organization Disability Assessment Schedule (WHODAS-2.0) is a widely used measure of functional impairment in cross-cultural studies [5]. The WHODAS-2.0 can be applied to any health condition and is recommended by the DSM-5 Disability Study Group as the best current measure of disability for research and routine clinical practice [6]. The WHODAS- 2.0 is based on the International Classification of Functioning, Disability and Health (ICF) [7]. In the ICF framework, functioning is interpreted as a dynamic interaction between health conditions and contextual factors [8]. Accordingly, the WHODAS-2.0 was developed to measure difficulty with daily activities and social participation [9] due to any health condition, including diseases, illnesses, injuries, mental or emotional problems and problems with alcohol or drugs [10, 11].

The WHODAS-2.0 has been used in population surveys, for monitoring individual patient outcomes in clinical practice and to assess the effectiveness of interventions in reducing disability [7]. The WHODAS 2.0 has been validated in several high income countries, including Germany and Portugal [11, 12], and middle income countries such as China [13]. The psychometric properties of WHODAS have been evaluated in a number of studies, including population samples and in people with a range of health conditions, including severe mental disorders (SMD) [3, 5, 11, 12, 14–16]. However, to our knowledge, there are no published studies on the validation of the WHODAS among people with SMD in a rural African setting.

The aim of the current study was, therefore, to evaluate the psychometric properties of the Amharic translated version of the WHODAS-2.0 among people with SMD in a rural African setting.

## Methods

### Setting and context of the study

The study was carried out around Butajira, Gurage Zone, Southern Nations, Nationalities and Peoples’ Region. Butajira is located 135 km south of Addis Ababa, the capital city of Ethiopia; it is a predominantly rural area, with farming being the main livelihood. Butajira is the site of a previous population-based epidemiological studies of SMD [17]. At the time of our study, mental health care services in the Butajira area were only available in a psychiatric nurse-led out-patient clinic at Butajira general hospital; however, a programme to train primary care workers to deliver mental health care and expand access is underway [18]. This service expansion is being evaluated through the TaSCS trial (Task-Sharing for the Care of Severe Mental Disorders in a low-income country) [19]. The current evaluation of the WHODAS was carried out as part of preparatory work to develop/adapt and culturally validate outcome measures for the TaSCS trial.

### Translation and technical and content validation of the WHODAS 2.0

The WHODAS 2.0 is available in different forms depending on the number of items (12 and 36 item versions), the mode of administration (self- vs. interviewer administered) and the respondent (patient, caregiver and clinician) [6, 7, 11, 12, 20]. The WHODAS presents list of health conditions with flash card #1 and response categories with flash card #2 visually. Card #1 helps respondents to visualize and easily understand the meaning of health conditions, what does having difficulty with an activity mean and the kinds of health conditions that the WHODAS can be used for. Card #2 aids the respondents to visualize and easily capture the response categories.

In this study, the Amharic 36 item patient version of the WHODAS 2.0 was used as a starting point for further optimizing the translation, and examining technical and content validity. This version was used previously in the Butajira area [21, 22]. For this version, forward and backward translation was done by four research assistants who are fluent Amharic speakers, trained at masters’ degree level and had experience of translating, adapting and using mental health measures.

As part of a free listing and pile sorting exercise carried out to identify potential items for a separate study to develop a contextual functioning scale [23], the relative importance of different items of the WHODAS-2.0 to the Butajira setting was explored. Six group discussions were conducted, three composed of men and the other three composed of women. Each group comprised six participants selected purposively from the community on the basis of their age (aged 18 years or above), living in the area for most of their life and their ability to express themselves well. Participants were asked ‘what are the tasks that men/women must do regularly to care for themselves? Their family? Their community?’ The facilitator then probed using items on the WHODAS after an exhaustive list of tasks had been generated spontaneously. Participants were asked whether the WHODAS items were relevant for the community.

Further exploration of the content validity of the WHODAS was carried out by administering the scale to a sample of people with SMD (*n* = 20; 10 males) and their caregivers (*n* = 20; 15 males) recruited from the psychiatric unit of Butajira general hospital. All participants were from rural areas and had educational level primary or less. People with SMD diagnosed by psychiatric nurses as having schizophrenia, bipolar disorder or major depressive disorder and their caregivers who were coming to the psychiatric clinic either for the first time or for follow-up appointment were consecutively recruited. Cognitive interviewing was used to identify any difficulty with understanding of individual items and response categories, as well as acceptability and burden of the whole scale. We then presented the findings to an expert panel composed of psychiatrists, psychologists, social workers and mental health researchers. Expert panel members were selected on the basis of their qualification and experience with adaptation and validation of mental health measures and their familiarity with the study setting. Expert panel members were working at the Addis Ababa University as researchers and faculty members at the of time the study. The panel members suggested how each problematic item should be rephrased to be more easily understood by the respondents, while retaining semantic equivalence with the original scale.

### Convergent and construct validation of the WHODAS 2.0

A facility-based cohort study was carried out to evaluate the sensitivity to change of the WHODAS-2.0. Internal consistency and convergent validity were assessed using the baseline sample.

### Sample

People with SMD presenting with new onset of illness or relapse of existing illness, and their caregivers, were recruited from the Butajira general hospital psychiatric clinic. For the purpose of determining the correlation between the BPRS-E and the WHODAS 2.0, a sample of *n* = 118 was calculated (to detect a correlation coefficient of 0.8 between the two continuous measures, with a margin of error of 0.1, alpha = 0.05 and power of 80%) [24]. However, we were able to manage to recruit 150 people with SMD and their caregivers (*n* = 150). In order to study the sensitivity to change of the WHODAS-2.0, a sample size of 90 people was required. This is to detect a standardized effect size of 0.6, with 80% power and alpha = 0.05 [25]. However, we were able to assess 84 of the required 90. That is a random sub-sample of 84 people with SMD and their caregivers (*n* = 84) were assessed at follow-up 6 weeks after the baseline assessment. We used the 6 weeks follow-up period because new cases with severe mental disorder begin to bring both symptomatic and functional improvement within 6 weeks of starting to take medication [26]. An additional 100 people with SMD and their caregivers (*n* = 100) to the 150 were recruited from the Butajira general hospital psychiatric clinic in order to give a total of *n* = 250 sample, which is adequate for conducting confirmatory factor analysis (CFA).

The inclusion criteria included DSM-IV diagnosis of schizophrenia, bipolar disorder or major depressive disorder with psychotic features made by psychiatric nurses, new onset or in acute relapse, age 18 years or over and able to attend for a follow-up appointment. Our reasons for conducting the study with these disorders are: 1) they are priority disorders in low and middle income countries as they are disabling and associated with human rights abuses 2) this study is nested in a bigger project looking at the impact of task sharing the care of people with these disorders [19]. The exclusion criteria were severe co-morbid physical health condition and substance dependence or abuse (as these may limit the participants’ ability to complete self-report measures).

### Measures

Data were collected by lay interviewers (*n* = 5; 3 males). All the five data collectors have diploma level educational qualification and have 15 years of field work experience employed in a long term mental health research project. Refresher training was given for one day to familiarize the data collectors with the new measures.

The Amharic version of the 36 item WHODAS 2.0 was administered. The full version of the WHODAS- 2.0 comprises 36 items in six domains [5]: understanding and communicating (6 items), getting around (5 items), self-care (4 items), getting along with others (5 items), activities at home, work and/or school (8 items) and participation in society (8 items). There are five response options for each item (none, mild, moderate, severe and extreme/cannot do). For each item, respondents are required to estimate the magnitude of their disability during the past 30 days. WHODAS 2.0 scores are computed for each domain by adding the item responses; a global score is also calculated from all the items [5]. A higher score indicates greater disability or worse functioning. The two best performing items from each domain were chosen for the 12 item version [14]. WHODAS- 2.0 has high internal consistency, moderate to good test-retest reliability [7] and good concurrent validity [5].

The Expanded version of the Brief Psychiatric Rating Scale (BPRS-E) was used to assess severity of clinical symptoms [27]. The BPRS-E is a 24-item observer-rated symptom scale covering four domains of symptoms of SMD (positive symptoms, negative symptoms, anxiety and depressive symptoms, and manic excitement or disorganization). The BPRS-E has been used widely to detect clinical improvement in response to an intervention [28] and has been used previously in Ethiopia [29]. Psychiatric nurses were trained in BPRS-E administration by a psychiatrist and practiced joint rating prior to the study.

A structured questionnaire was used to collect data on the gender, age, education, marital status and relative wealth of both people with SMD and their caregivers. The diagnosis of each patient was extracted from the clinical notes.

### Data analysis

Convergent validity (comparing the WHODAS 2.0 with severity of symptoms) was assessed by calculating Pearson’s correlation coefficient (*r*). Internal consistency of each of the domain and overall WHODAS 2.0 items was assessed by calculating Cronbach’s alpha.

In order to evaluate the sensitivity to change of the WHODAS 2.0, both internal and external responsiveness were determined in line with recommended practice [30]. Internal responsiveness is the change in a measure over time and was evaluated by a paired sample t-test, effect size (ES), calculated as change in mean divided by standard deviation of the baseline score, and the standardized response mean (SRM), calculated by dividing the change in mean score by the standard deviation of the change scores (Δ mean / Δ SD). External responsiveness is the extent to which change in the index measure (WHODAS 2.0) corresponds to change in an external, reference measure (the BPRS-E) [30]. Spearman rank order correlation of the change scores from the two measures was computed to determine external responsiveness to change.

CFA was carried out to test whether the six domains of the 36 item WHODAS- 2.0 and the one-dimensional nature of the 12 item WHODAS are applicable in the rural Ethiopian context. We conducted a second order CFA to test the structure of the 36 item WHODAS-2.0. The first order factors were the six domains, each containing four to eight items and the second order factor was the general disability factor. Goodness of fit was assessed with the following indices: χ^2^ test, acceptable if χ^2^/*df* is less than 3.0 [31]; Comparative Fit Index (CFI), acceptable if its value ≥0.95 [32]; Tucker-Lewis Index (TLI), acceptable if its value exceeds 0.90 [32]; and root mean square error of approximation (RMSEA), acceptable if the value is close to 0.06 [33].

### Ethical considerations

The study was approved by the Institutional Review Board of the College of Health Sciences, Addis Ababa University. Written informed consent was obtained from most of the service users and all of the caregivers. For a few service users, who were acutely unwell, we either obtained permission from their guardians or obtained written consent at the follow-up assessment after their condition had improved.

## Results

### Adaptation and content validity

For details of the difficulties identified for each item and the resulting amendments, see Additional file 1. Iterative adjustments were made to the Amharic translations, including the addition of examples to items asking about broad and abstract experiences, and replacing less relevant and uncommon concepts to the setting with equivalent but local experiences. Misunderstanding of items was more apparent in people residing in rural areas who had no formal education. However, there were questions that were difficult to understand even by those who were educated, urban and native Amharic speakers.

Items in the cognition domain were generally found to be abstract and difficult to understand. Some respondents only listened to the first component of a multi-clause question, ignoring or forgetting the other aspects. In the mobility domain, the item “walking a long distance such as a kilometer [or equivalent]” was problematic as this distance is not considered to be a long distance in the study context. People in rural Ethiopia walk long distances almost every day for work or social activities, as there is limited access to transportation. Two of the items in the self-care domain (“eating” and “staying by yourself for a few days”) were not considered to be relevant, as most people lived with extended family and there was no tradition of independent living. Indeed, staying alone was considered to be dysfunctional, related to being depressed or wanting to be alone. We improved the relevance of this item by training interviewers to ask a hypothetical question; that is whether or not the person would be able to stay by themselves for a few days if they were left alone. The item “eating” was modified to ask about difficulty with properly feeding oneself.

Many of the items in the “getting along with people” domain were initially problematic. For instance, the Amharic translation of the item “sexual activities” was found to be offensive and unacceptable, especially for people who were single, widowed and separated. Caregivers were embarrassed to be asked about the sexual activities of their family member and had little knowledge of the person’s private life. For the item “sexual activities”, we changed the Amharic translation to ask about romantic relationships. Nobody understood correctly the items “how much of a problem did you have because of barriers or hindrances in the world around you?” and “how much of a problem did you have living with dignity because of the attitudes and actions of others?” These items required simplification and addition of examples.

Items in the household activities domain were mostly straightforward to understand. However, respondents requested examples of household activities. It was also difficult for some respondents to distinguish among items “doing important household tasks well, getting all the household work done and getting household work done as quickly as needed.” A similar problem was observed when these questions referred to work or school. Some male respondents were of the view that it was not their responsibility to accomplish household activities.

### Technical validity

The visual presentation of the list of health problems, definition of difficulty to accomplish a task and response categories (cards # 1 and # 2) were helpful prompts for respondents. It was difficult for almost all respondents to answer questions related to “for how many days were these difficulties present in the past 30 days?”

### Psychometric properties of WHODAS 2.0

A total of 150 people with SMD and 150 caregivers participated in the facility-based cohort study to determine convergent validity and sensitivity to change of the Amharic adapted version of the WHODAS-2.0. A random sub-sample of 84 people was followed up out of the intended 90. An additional 100 people with SMD and their caregivers (*n* = 100) were recruited at baseline for CFA analysis, giving a final sample size of 250. The socio-demographic characteristics of the participants are presented in Table 1.

**Table 1:** Socio-demographic characteristics of participants

Characteristics		Validation study		Additional sample for CFA	
		Service users (*n* = 150)	Caregivers (*n* = 150)	Service users (*n* = 100)	Caregivers (*n* = 100)
		*N* (%)	*N* (%)	*N* (%)	*N* (%)
Sex	Male	81 (54.0)	121 (80.7)	71 (71.0)	83 (83.0)
	Female	69 (46.0)	28 (18.7)	29 (29.0)	17 (17.0)
Age (years)	Mean (SD)	30.42 (10.04)	35.09 (12.14)	33.05 (13.34)	34.95 (12.23)
Ethnicity	Gurage	60 (40.0)	58 (38.7)	48 (48.0)	47 (47.0)
	Siltie	80 (53.3)	82 (54.7)	42 (42.0)	43 (43.0)
	Amhara	1 (0.7)	2 (1.3)	2 (2.0)	2 (2.0)
	Oromo	2 (1.3)	1 (0.7)	2 (2.0)	2 (2.0)
	Other	7 (4.7)	7 (4.7)	6 (6.0)	6 (6.0)
Religion	Orthodox	27 (18.0)	25 (16.7)	19 (19.0)	19 (19.0)
	Muslim	118 (78.7)	120 (80.0)	76 (76.0)	78 (78.0)
	Protestant	5 (3.3)	5 (3.3)	5 (5.0)	3 (3.0)
Marital status	Single	53 (35.3)	34 (22.7)	45 (45.0)	27 (27.0)
	Married	81 (54.0)	112 (74.7)	48 (48.0)	72 (72.0)
	Divorced	4 (2.7)	0 (0.0)	6 (6.0)	1 (1.0)
	Separated	7 (4.7)	0 (0.0)	0 (0.0)	0 (0.0)
	Widowed	5 (3.3)	3 (2.0)	1 (1.0)	0 (0.0)
Educational level	Can’t read and write	57 (38.0)	34 (22.7)	27 (27.0)	16 (16.0)
	Read and write only	41 (27.3)	57 (38.0)	29 (29.0)	25 (25.0)
	Primary	40 (26.7)	42 (28.0)	28 (28.0)	32 (32.0)
	Secondary	10 (6.7)	10 (6.7)	12 (12.0)	17 (17.0)
	Postsecondary	2 (1.3)	6 (4.0)	4 (4.0)	10 (10.0)
Occupation	Farming	77 (51.3)	109 (72.7)	54 (54.0)	56 (56.0)
	Trading	12 (8.0)	13 (8.7)	8 (8.0)	14 (14.0)
	Government employee	1 (0.7)	8 (5.3)	3 (3.0)	11 (11.0)
	Student	9 (6.0)	6 (4.0)	5 (5.0)	5 (5.0)
	Housewife	27 (18.0)	12 (8.0)	13 (13.0)	8 (8.0)
	Have no job	22 (14.7)	1 (0.7)	17 (17.0)	6 (6.0)
	Other	2 (1.3)	1 (0.7)	0 (0.0)	0 (0.0)
Relative wealth	Less	67 (44.7)	42 (28.0)	32 (32.0)	13 (13.0)
	More	4 (2.7)	4 (2.7)	5 (5.0)	4 (4.0)
	Equal	79 (52.7)	104 (69.3)	63 (63.0)	83 (83.0)
Diagnosis	Schizophrenia	68 (45.3)	-	57 (57.0)	-
	Bipolar disorder	41 (27.3)	-	24 (24.0)	-
	Major depressive disorder	41 (27.3)	-	19 (19.0)	-

*CFA* confirmatory factor analysis, *n* total number of respondents, *N* number of respondents in a given category of background characteristics

### Internal consistency

Internal consistency of the items in the total scale and of the domains was either very good or excellent (Cronbach’s alpha ranging from 0.82 to 0.98). Cronbach’s alpha coefficients for sub-scales and the overall WHODAS ranged between 0.88 and 0.98 among service users and 0.82 to 0.99 among caregivers.

### Convergent validity

Both at baseline and follow-up, the scores of the overall scale and all domains of the WHODAS 2.0 were found to have a positive correlation with the BPRS-E scores (Table 2), although weaker at baseline and higher in the caregiver sample. Correlation coefficients ranged from 0.13 to 0.22 among service users and 0.20 to 0.34 among caregivers at baseline, and from 0.29 to 0.51 among service users and 0.40 to 0.53 among caregivers at follow-up.

**Table 2:** Pearson’s correlation coefficients between BPRS-E and WHODAS-2.0 (*N* = 150)

	Service users		Caregivers	
WHODAS sub-scale	Baseline	Follow-up after 6 weeks	Baseline	Follow-up after 6 weeks
Cognition	0.18	0.50	0.34	0.50
Mobility	0.13	0.29	0.20	0.40
Self-care	0.22	0.51	0.32	0.53
Getting along	0.22	0.43	0.24	0.53
Household activities	0.16	0.43	0.27	0.42
Work/school	0.13	0.43	0.26	0.43
Participation	0.22	0.45	0.27	0.49
Overall WHODAS	0.21	0.47	0.32	0.52

### Sensitivity to change

The mean scores of the overall WHODAS and of all the domains were reduced after 6 weeks of treatment for new or acutely relapsed cases and found to be statistically significant among both service users (Table 3) and caregivers (Table 4). However, the effect sizes and the standardized response means were small among service users (ranging from 0.17 to 0.35) and moderate among caregivers (ranging from 0.14 to 0.57). For both service users and caregivers, the lowest sensitivity to change was in the mobility sub-scale while the largest sensitivity to change was in the work/school sub-scale.

**Table 3:** Internal sensitivity to change (*N* = 84 service users)

	Baseline		Follow-up		Difference		ES	SRM
WHOADS sub-scale	Mean	SD	Mean	SD	Mean	SD		
Cognition	18.26	8.13	15.40	7.67	2.86	8.77	0.35	0.33
Mobility	12.24	5.89	11.23	5.64	1.01	5.86	0.17	0.17
Self-care	9.27	5.09	8.08	4.57	1.19	4.75	0.23	0.25
Getting along	13.89	6.28	12.10	6.23	1.80	6.77	0.29	0.27
Household activities	12.74	5.70	11.10	5.59	1.64	5.84	0.29	0.28
Work/school	12.93	5.75	11.04	5.56	1.89	5.80	0.33	0.33
Participation	24.54	10.21	22.10	9.80	2.44	9.38	0.24	0.26
Overall WHODAS	103.87	42.14	91.04	41.45	12.83	40.51	0.30	0.32

*SD* standard deviation, *ES* effect size, *SRM* standardized response mean

**Table 4:** Internal sensitivity to change (*N* = 84 caregivers)

	Baseline		Follow-up		Difference		ES	SRM
WHODAS sub-scale	Mean	SD	Mean	SD	Mean	SD		
Cognition	20.36	7.36	17.02	7.82	3.33	8.42	0.45	0.40
Mobility	12.52	5.78	11.69	5.74	0.83	5.90	0.14	0.14
Self-care	10.85	4.93	8.71	4.83	2.13	4.67	0.43	0.47
Getting along	15.77	6.03	12.69	6.41	3.08	6.61	0.51	0.47
Household activities	14.68	4.77	12.18	5.57	2.50	5.77	0.52	0.43
Work/school	14.88	4.74	12.17	5.64	2.71	5.72	0.57	0.47
Participation	27.82	8.59	23.81	9.40	4.01	8.88	0.47	0.45
Overall WHODAS	116.88	37.29	98.27	41.30	18.61	39.25	0.50	0.47

*SD* standard deviation, *ES* effect size, *SRM* standardized response mean

Spearman’s correlation coefficients between the change scores of the WHODAS and BPRS-E showed that the scores on the two measures co-vary together. However, the correlation coefficients were either small or moderate, ranging from 0.13 to 0.32 among service users and 0.25 to 0.40 among caregivers (Table 5).

**Table 5:** External sensitivity to change (*N* = 84)

	Spearman’s correlation coefficient	
WHODAS sub-scale	Service users	Caregivers
Understanding	0.32	0.39
Mobility	0.13	0.33
Self-care	0.20	0.27
Getting along	0.27	0.25
Household activities	0.20	0.35
Work/school	0.24	0.34
Social participation	0.20	0.28
Overall WHODAS	0.26	0.40

### Confirmatory factor analysis

**Fig. 1:**
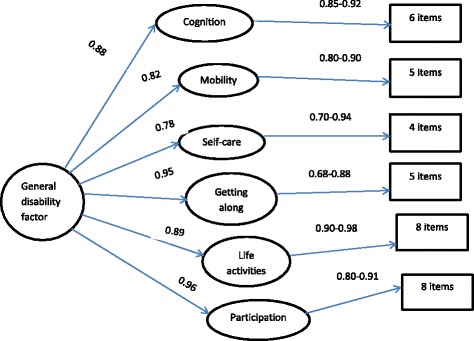
Second order confirmatory factor analysis of the WHODAS-2.0, 36 item version

The second order factor, the general disability factor, had factor loadings ranging from 0.78 (self-care) to 0.96 (participation) (Fig. 1). Among the six first order factors, the items of Domain 5 (life activities) had the largest factor loadings (0.90–0.98), whereas the items of Domain 4 (getting along with people) had the smallest factor loadings (0.68–0.88). The goodness of fit indices were close to, but outside of, the acceptable ranges (χ^2^/df = 3.46; CFI = 0.89; TLI = 0.88 and RMSEA = 0.099)*.* Both factor loadings and goodness of fit indices were similar for the service user and caregiver data.

The item-factor loadings of the one factor model of the 12 item WHODAS were high, ranging from 0.71 to 0.89 among service users and 0.50 to 0.88 among caregivers. There were two items with factor loadings below 0.60 among caregivers, which were both in the mobility sub-scale. The goodness of fit indices were a little outside of the acceptable ranges, both among service users and caregivers (χ^2^/df = 10.13; TLI = 0.79; CFI = 0.82 and RMSEA = 0.19 among service users and χ^2^/df = 7.68; TLI = 0.80; CFI = 0.84; and RMSEA = 0.16 among caregivers).

## Discussion

In this study, we improved the Amharic translation of the 36 item WHODAS for a rural Ethiopian context, while retaining semantic equivalence with respect to the original scale, and assessed psychometric properties in a sample of people with SMD. We found that items in the mobility and self-care domains were the easiest and that items in the participation domain were the most difficult to understand. There was evidence that the experiences assessed by the WHODAS items and the way they are framed favor educated and urban respondents. Our improved version of the WHODAS had excellent internal consistency, and good evidence for convergent and construct validity and responsiveness to change in clinical state of people with SMD. While improving the Amharic translation, we keep the essence of each item equivalent to the original English version and did not delete or add any item. We sought to maintain semantic, content, technical and other aspects of equivalence throughout our exercise of improving the Amharic version of the WHODAS-2.0. Thus, we argue that the improved Amharic version of the WHODAS that we used in this study can be used in other similar settings and that it would ensure comparability of data.

We found that the majority of the respondents were not able to remember the exact number of days that they were functionally impaired. Because of this we were not able to use the data from these items for any analysis. We were not able to identify other published studies from a rural African context with which to compare our findings regarding the difficulty of the WHODAS items. A few studies from LAMICs such as rural China [34, 35] found items which were not applicable to the context, with similarities to our findings (e.g. with sensitivity of the item asking about “sexual activities” in both contexts). Overall, our findings suggest that the WHODAS items require rigorous adaptation (forward and backward translation, cognitive interviewing, expert consensus and pilot testing) to ensure that they have content validity while retaining content equivalence in settings such as rural Ethiopia.

We found that the overall WHODAS has excellent internal consistency (0.98) and the internal consistency of the sub-scales was either very good or excellent, ranging from 0.82 to 0.98. These values are in line with findings from many other previous studies conducted in samples of people with a range of different health conditions [5, 7, 15, 16], but higher than a few other studies [11].

Our study shows that the score of the overall WHODAS and the sub-scale scores have a positive correlation with symptom severity scores, both at baseline and follow-up. The correlation coefficients were higher at follow-up than at baseline, which may be explained by the distribution of scores. There was low variability of both the WHODAS and BPRS-E scores at baseline (scores were consistently high) as we included new or acutely relapsed cases. Our finding that symptom severity and disability scores are positively correlated is consistent with previous studies [21, 36, 37]. Moreover, we found that the correlation between symptom severity and disability scores was either weak or moderate which was consistent with our expectations. In our previous qualitative study [38], we found that functional impairment in people with SMD is associated not only with illness symptoms, but also with other personal, family, social and economic factors. The scores of the overall WHODAS and the sub-scales were found to have positive and strong correlations with the overall score and sub-scale scores of the BFS, a locally developed functioning measure for people with SMD. This is important evidence to support the convergent validity of the WHODAS-2.0 in the rural African context for people with SMD.

Our study shows that the WHODAS-2.0 has the ability to detect small changes over time. We found statistically significant mean changes in disability scores after 6 weeks treatment of new and acutely relapsed cases. However, the effect sizes and SRM were small among service users and moderate among caregivers. The change in the overall WHODAS scores in terms of effect size was 0.30 among service users and 0.50 among caregivers. The smaller effect sizes using the service user responses to the WHODAS may be due to under-reporting of functional impairment by people with psychosis [39], both at baseline and follow-up. Since we included new or acutely relapsed cases of schizophrenia, bipolar disorder and depression with psychotic features, the service users may lack capacity to accurately evaluate their functional status [40]. For both the service user and caregiver WHODAS responses, we obtained smaller effect sizes compared to those obtained in other previous studies [5, 7]. Nonetheless, there was a positive and statistically significant correlation between the change scores of symptom severity and disability, indicating that change in symptom severity is accompanied by change in disability scores.

The WHODAS sub-scale with the smallest effect size was mobility (0.17 among service users and 0.14 among caregivers). Other sub-scales had effect sizes ranging from 0.23 to 0.35 among service users and 0.43 to 0.57 among caregivers. This finding is expected and consistent with previous studies [5]. The mobility sub-scale had the smallest mean value at baseline; it is also expected that mental health problems have more impact on occupational and social functioning rather than on mobility [41].

The high factor loadings and the goodness of fit indices indicated that the six domain structure and the global score of the 36 item WHODAS −2.0 and the global score of the 12 item WHODAS could be used in this rural African setting. Nevertheless, none of the indicators of goodness of fit were within the recommended ranges. CFA modification indices suggested that the goodness of fit may be improved if some items from some domains were allowed to correlate. Overall, accepting the original structure proposed by developers would improve comparability with past and ongoing studies on the WHODAS. Our findings regarding the factor structure of both the 36 -item and the 12- item WHODAS are more or less similar with previous studies, both from specific populations [15, 16, 20, 42] and from modified versions [34, 35, 43].

An important finding was that the 36 -item and the 12 -item WHODAS have similar psychometric properties, including internal consistency, convergent validity, responsiveness to change and factor structure. However, the 12 item WHODAS was superior, both in terms of understandability and contextual relevance. Previous studies found that the 12 item WHODAS is feasible and acceptable [12]; and is similar in terms of psychometric properties to the full version [42]. These findings all indicate that the single factor 12 item WHODAS is the preferred version in this rural low income country setting.

Using a cross-cultural measure of disability is an advantage for the purpose of comparing research findings across different cultural contexts. Our study shows that WHODAS-2.0 could be used as an outcome measure in different cultural contexts. However, there are items in the WHODAS which are difficult to understand and are not relevant to rural African contexts. Male respondents were generally less interested to respond to items in the household activities domain believing that women are totally responsible for accomplishing all types of tasks at home. This study highlights the importance of also using a functioning measure that is developed based on locally relevant tasks in rural African setting, such as the Butajira Functioning Scale (BFS) [23]. Recent attempts to develop local, health condition specific functioning instruments [38, 44] have demonstrated that it is possible to develop measures that are easy and quick to administer and psychometrically sound with items that are contextually relevant and acceptable.

There are recent initiatives in LAMICs, including Ethiopia, to scale up evidence-based packages of mental health care [19] through task sharing and integrating the service into primary care. This is recommended by the WHO in the Mental Health Gap Action Program (mhGAP) [45] and endorsed by the Federal Ministry of Health of Ethiopia. It is necessary to evaluate the impact of scaling up mental health care on functional outcomes, in addition to clinical outcomes. For this purpose, there is a need for a contextually relevant, but internationally comparable, and validated measure. Our findings indicate that adapted versions of the WHODAS can meet this need.

This is the first study, to our knowledge, to examine the psychometric properties of the WHODAS-2.0 among people with SMD in a rural African context. We followed rigorous translation and validation procedures. However, the following limitations need to be taken into consideration. We determined responsiveness to change after 6 weeks treatment of people with new onset or in relapse, but people with SMD may not experience functional improvement within this short time period [26]. Due to logistical challenges, we did not assess test-retest reliability. The sample size for CFA was relatively small, although within recommended limits. The studies reported in this paper were conducted in a rural area of Ethiopia and are not likely to be generalisable to urban areas of Ethiopia. Although the adaptation and validation was conducted in people with severe mental disorders, we did not change the content of items and expect that the findings could be generalizable to other mental disorders and health conditions.

## Conclusions

The WHODAS-2.0 has acceptable psychometric properties as a cross-cultural measure of functional impairment, with careful translation and modification of problematic items. Future research should focus on test-retest reliability, sensitivity to change with longer duration of follow-up and item response theory analysis.

## Additional file

Additional file 1: Summary of the issues observed and the amendments made in optimizing the translation and adaptation of the WHODAS-2.0 items. (DOCX 40 kb)

## Abbreviations

BFS: Butajira Functioning Scale
BPRS: Brief Psychiatric Rating Scale
CFA: Confirmatory factor analysis
CFI: Comparative fit index
DSM: Diagnostic and Statistical Manual for Mental Disorders
ES: Effect size
ICF: International Classification of Functioning, Health and Disability
LAMICs: Low and middle income countries
mhGAP: Mental Health Gap Action Program
RMSEA: Root mean square error of approximation
SMD: Severe mental disorders
SRM: Standardized response mean
TASCS: Task- sharing for the care of severe mental disorders in a low-income country setting
TLI: Tucker-Lewis index
WHO: World Health Organization
WHODAS: World Health Organization Disability Assessment Schedule
