# Supplementary material for: Validation of the World Health Organization Disability Assessment Schedule in people with severe mental disorders in rural Ethiopia
Source: Health Qual Life Outcomes. 2017 Apr 5;15:64. doi: 10.1186/s12955-017-0647-3 (PMC5382515; doi:10.1186/s12955-017-0647-3)
Supplement: Additional file 1: — Summary of the issues observed and the amendments made in optimizing the translation and adaptation of the WHODAS-2.0 items. (DOCX 40 kb) [file 12955_2017_647_MOESM1_ESM.docx]

**Additional file 1: Summary of the issues observed and the amendments made in the adaptation of the WHODAS-2.0 items**

| Original item in English | Issue/problem observed | Amendment/intervention | Adapted item in Amharic |
| --- | --- | --- | --- |
| Response categories | - There was a tendency to respond to each item dichotomously as “able to do the task” or “unable to do the task” - Difficulty to recall all of the response categories as the interview progresses | - Training of the interviewers to probe the respondents to catch all of the response categories and remind them while asking each item - We prepare flash cards and trained interviewers to use these as aids for respondents to easily understand and recall the response categories |  |
| Meaning of health problems and difficulty to do a task | - Difficulty understanding the descriptions about what “health problems” and “difficulty to do a task” mean - Forgetting these descriptions as the interview progresses | - Improving the translation in an expert consensus meeting - We prepare flash cards and trained interviewers as to how they could use these as aids for respondents to easily understand what does health problems and difficulty to do a task mean |  |
| **Cognition** | | | |
| Concentrating on doing something for ten minutes? | - Tendency to focus on ability to do something, not on concentration - Focusing on the concentration, forgetting concentrating on doing a task - Unable to know the Amharic translation of “concentrating.” | - Changing the Amharic translation of “concentrating” into an easier equivalent word - Connecting concentrating and doing something clearly in the Amharic version - Training interviewers to give emphasis on the connection between concentrating and doing something | በሚሰሩት ስራ ላይ **ለጥቂት ጊዜ (ለ10 ደቂቃ) ያህል ትኩረት ማድረግ** ምን ያህል ይቸግርዎት ነበር? |
| Remembering to do important things? | - There was a tendency to understand the question simply as remembering things, not to do things - That is understanding the question as capacity to recall or absence of problem of forgetting | - Connecting remembering and doing things clearly in the Amharic version - Training interviewers to give emphasis on the connection between remembering and doing things | **ማድረግ የሚፈልጓቸውን ነገሮች አስታውሰው ለማድረግ** ምን ያህል ይቸግርዎት ነበር? |
| Analyzing and finding solutions to problems in day-to-day life? | - Tendency to understand the question as solving problems related to the illness - Relating the question with work or thinking - This question was generally found to be complex, abstract and very difficult to understand | - Improving the translation, without changing the intention of the question, to simplify the item a little bit - Taking experts’ suggestion regarding how the clarity of the item can be improved - Generally one of the problematic items even after interventions | **በእለት ተእለት ሕይወትዎ ውስጥ የሚገጥምዎትን ችግሮች መንስኤ ለመለየትና መፍትሄ ለመፈለግ** ምን ያህል ይቸግርዎት ነበር? |
| Learning a new task, for example, learning how to get to a new place? | - A few respondents said that they did not experience a new task to learn in the last 30 days | - Interviewers were trained to encourage respondents to recall a task they have learned in the last thirty days - Some examples of possible new tasks that could be learned in the area were given | **አዲስ ነገር ወይም ስራ ለመማር** ምን ያህል ይቸግርዎት ነበር? (ለምሳሌ፡ የእርሻ ስራ፤ ባልትና፤ የእጅ ስራ ወዘተ…) |
| Generally understanding what people say? | - There was no issue on this item |  | **በአጠቃላይ ሰዎች የሚሉትን ለመረዳት** ምን ያህል ይቸግርዎት ነበር? |
| Starting and maintaining a  conversation? | - This item was well understood by the respondents - The Amharic translation had a sense of maintaining a conversation once it is started - Starting a conversation was not highlighted in the translation | - The translation was improved highlighting both starting and maintaining a conversation - Interviewers were trained to remind respondents to focus both on starting and maintaining a conversation | **ከሰዎች ጋር ንግግር ለመጀመርና እስከመጨረሻው ለመቆየት** ምን ያህል ይቸግርዎት ነበር? |
| **Mobility** | | | |
| Standing for long periods such as 30  minutes? | - There was no issue on this item |  | ረዘም ላለ ጊዜ **ቆሞ መቆየት** ምን ያህል ይቸግርዎት ነበር? (ለምሳሌ፡ ለግማሽ ሰዓት) |
| Standing up from sitting down? | - There was no issue on this item |  | **ከተቀመጡበት ለመነሳት** ምን ያህል ይቸግርዎት ነበር? |
| Moving around inside your home? | - There was no issue on this item |  | **እቤትዎ ውስጥ መዘዋወር** ምን ያህል ይቸግርዎት ነበር |
| Getting out of your home? | - There was no issue on this item |  | **ከቤትዎ ለመውጣት** ምን ያህል ይቸግርዎት ነበር? |
| Walking a long distance such as a kilometre [or equivalent]? | - Walking for a kilometer or for 15 minutes was not considered as a long walk in the setting as people are to travel long for work or social activites | - We did not make amendment for this item as it would change its original sense - Became one of the problematic items in the final adapted version | **የተወሰነ ርቀት መንገድ ለመጓዝ** ምን ያህል ይቸግርዎት ነበር? (ለምሳሌ፡ የሩብ ሰዓት መንገድ ወይም አንድ ኪሎ ሜትር) |
| **Self-care** | | | |
| Washing your whole body? | - There was no issue on this item |  | **ሰውነትዎን መታጠብ** ምን ያህል ይቸግርዎት ነበር? |
| Getting dressed? | - There was no issue on this item |  | **ልብስዎትን ለመልበስ** ምን ያህል ይቸግርዎት ነበር? |
| Eating? | - Some respondents understood this item as appetite, few others relate it with availability and quality of the food they eat | - We changed the sense of the Amharic translation to make it clear that the item is asking about difficulty to properly feeding oneself | **ምግብ ለመመገብ** ምን ያህል ይቸግርዎት ነበር |
| Staying by yourself for a few days? | - This didn’t happen for most of the respondents - Seems inappropriate for a culture where extended family is common - Some consider this as a problem by itself - They relate it with being depressed or wanting to be alone - Family members don’t allow them to stay by themselves even for a day | - This item was very difficult to amend as the experience is not existing in the setting - We trained the interviewers to ask respondents a hypothetical kind of question - Whether or not they would be able to stay by themselves for a few days if they were left alone | ያለሰው ብቻዎትን ለተወሰኑ ቀናት መቆየት ሲኖርብዎት **ብቻዎትን መቆየት** ምን ያህል ይቸግርዎት ነበር? |
| **Getting along with people** | | | |
| Dealing with people you do not know? | There was no issue on this item |  | **ከዚህ በፊት ከማያውቋቸው ሰዎች ጋር ለመጀመሪያ ጊዜ ለመግባባት** ምን ያህል ይቸግርዎት ነበር? |
| Maintaining a friendship? | There was no issue on this item |  | ከአንድ ሰው ጋር **በጓደኝነት ለብዙ ጊዜ መቆየት** ምን ያህል ይቸግርዎት ነበር? |
| Getting along with people who are close to you? | - It was difficult for a few respondents to know who these people are (people who are close to someone) | - We include in the item some examples of people who are close to someone (family members, relatives, close friends) | ከቤተሰቦችዎ፤ ከዘመዶችዎ እና ከቅርብ ጓደኞችዎ ጋር **ተግባብቶ መኖር** ምን ያህል ይቸግርዎት ነበር? |
| Making new friends? | - A few respondents said that they were not acquainted with a new person in the last 30 days - In rural areas and small towns, there may not be opportunity to access new friends within 30 days | - It was very difficult to make amendment on this item as the experience was not relevant to a few of the respondents; so was one of the problematic item | **አዲስ ጓደኝነት መጀመር** ምን ያህል ይቸግርዎት ነበር? |
| Sexual activities? | - This question was a little bit sensitive, and for some respondents offensive and unacceptable - It is also not applicable to some respondents (single, widowed, separated) - It was even embarrassing for caregivers for being asked about the sexual activities of their family member | - As per the suggestion of the expert committee, we changed the Amharic translation to be a bit broad having the sense of making romantic relationship with opposite sex - This was more inclusive and less sensitive and offensive | **ከተቃራኒ ፆታ ጋር የፍቅር ግንኙነት ማድረግ** ምን ያህል ይቸግርዎት ነበር? |
| **Life activities (house hold activities)** | | | |
| Taking care of your household responsibilities? | - Household responsibilities were found to be very general and respondents needed a few examples - A few men respondents understood the item as doing domestic tasks such as cooking and washing clothes and said that they are to be accomplished by women | - We trained interviewers to give a few common examples of household responsibilities in the setting for those who needed - We modified the translation to make the item more inclusive beyond domestic tasks | **የቤትና የግቢ ውስጥ ስራዎችንና ሌሎች ኃላፊነቶችን** **መወጣት** ምን ያህል ይቸግርዎት ነበር? |
| Doing your most important household tasks well? | - The same issue as the item “taking care of household responsibilities - Difficulty to make distinction among doing household tasks well, getting all the household work done and getting the work done as quickly as needed | - We trained interviewers to give a few common examples of household responsibilities in the setting for those who needed - We improved the translation and tried to make the three items clearly distinct | በጣም አስፈላጊ የሚሏቸውን የቤትና የግቢ ውስጥ ስራዎች **በጥሩ ሁኔታ** መስራት ምን ያህል ይቸግርዎት ነበር? |
| Getting all the household work done that you needed to do? | - The same issue as the item “taking care of household responsibilities - Difficulty to make distinction among doing household tasks well, getting all the household work done and getting the work done as quickly as needed | - We trained interviewers to give a few common examples of household responsibilities in the setting for those who needed - We improved the translation and tried to make the three items clearly distinct | መስራት ያለብዎትን የቤትና የግቢ ውስጥ ስራዎች **ሁሉንም** ሰርቶ ለመጨረስ ምን ያህል ይቸግርዎት ነበር? |
| Getting your household work done as quickly as needed? | - The same issue as the item “taking care of household responsibilities - Difficulty to make distinction among doing household tasks well, getting all the household work done and getting the work done as quickly as needed | - We trained interviewers to give a few common examples of household responsibilities in the setting for those who needed - We improved the translation and tried to make the three items clearly distinct | የቤትና የግቢ ውስጥ ስራዎችን **በሚፈልጉት ፍጥነት** ለመስራት ምን ያህል ይቸግርዎት ነበር? |
| **Life activities (work or school activities)** | | | |
| Your day-to-day work/school? | - There was no any issue on this item |  | የእለት ተዕለት **ስራዎትን ወይም ትምህርትዎትን** ለማከናወን ምን ያህል ይቸግርዎት ነበር? |
| Doing your most important work/school tasks well? | - The same issue as items on household activities - That is there was difficulty to make distinction among doing your work or school tasks well, getting all the work done that you need to do and getting your work done as quickly as needed | - We improved the translation and tried to make the three items clearly distinct | በጣም አስፈላጊ የሚሉትን ስራ ወይም ትምህርት **በጥሩ ሁኔታ** መስራት ምን ያህል ይቸግርዎት ነበር? |
| Getting all the work done that you need to do? | - The same issue as items on household activities - That is there was difficulty to make distinction among doing your work or school tasks well, getting all the work done that you need to do and getting your work done as quickly as needed | - We improved the translation and tried to make the three items clearly distinct | መስራት ያለብዎትን ስራ ወይም ትምህርት **ሁሉንም ሰርቶ ለመጨረስ** ምን ያህል ይቸግርዎት ነበር? |
| Getting your work done as quickly as needed? | - The same issue as items on household activities - That is there was difficulty to make distinction among doing your work or school tasks well, getting all the work done that you need to do and getting your work done as quickly as needed | - We improved the translation and tried to make the three items clearly distinct | ስራዎትን ወይም ትምህርትዎትን **በሚፈልጉት ፍጥነት** ለመስራት ምን ያህል ይቸግርዎት ነበር? |
| **Participation** | | | |
| How much of a problem did you have joining in community activities (for example, festivities, religious or other activities) in the same way as anyone else can? | - There was no issue on this item |  | **በማሕበራዊ እንቅስቃሴ ውስጥ** (ለምሳሌ፡ አመት በዓል፤ ድግስ፤ ለቅሶ፤ እድር፤ ሊቃ ወዘተ…) ልክ እንደሌላው ሰው መሳተፍ ምን ያህል ይቸግርዎት ነበር? |
| How much of a problem did you have because of barriers or hindrances in the world around you? | - Almost all respondents did not understand this question correctly. - This item was difficult to understand even by respondents who were educated and urban - It is too long, complex and abstract | - We tried to improve the translation and make some adaptation by including examples of barriers and hindrances to improve its clarity and relevance - But, it was one of the problematic items in the final adapted version of the scale | እንደ አድሎና መገለል እና ሌሎችም በአካባቢዎ ባሉ **መሰናክሎችና ምቹ ያልሆኑ ሁኔታዎች** ምክንያት ምን ያህል ችግር ገጠመዎት? |
| How much of a problem did you have living with dignity because of the attitudes and actions of others? | - Almost all respondents could not understand this question correctly - Even better educated and respondents from urban areas didn’t understand what the question is about - It was very difficult for respondents to understand the meaning of “living with dignity” - The item is too long, complex and abstract | - We tried to find a better translation for the concept of “living with dignity” in the expert consensus meeting - We tried to make some adaptation without changing the initial intent of the item - Still, it was one of the problematic items in the final version of the scale | ሰዎች ለእርስዎ ባላቸው መጥፎ አመለካካትና ተገቢ ያልሆኑ ድርጊቶች የተነሳ **በሰው ተከብረው** ለመኖር ምን ያህል ተቸገሩ? |
| How much time did you spend on your health condition or its consequences? | - There was no issue on this item |  | ለሕመምዎ መፍትሄ ለማግኘት፤ ጠያቂ ለማነጋገር፤ ስለሕመምዎ ለሌሎች ለማስረዳት **ምን ያህል ጊዜ አጥፍተዋል**? |
| How much have you been emotionally affected by your health condition? | - There was no issue on this item |  | በጤና ችግርዎ ወይም በሕመምዎ ምክንያት **ስሜትዎ ምን ያህል ተረብሿል**? |
| How much has your health been a drain on the financial resources of you or your family? | - It was difficult to understand the Amharic translation of drain - Very few respondents asked for examples of financial resources | - We were able to find a better translation for the word “drain” in the expert consensus meeting - We trained interviewers to give some examples of financial resources for those who needed | የጤና ችግርዎ ወይም ሕመምዎ የእርስዎንና የቤተሰብዎን **ሀብትና ንብረት ምን ያህል አራቆተ**? |
| How much of a problem did your family have because of your health problems? | - There was no issue on this item |  | በእርስዎ የጤና ችግር ወይም በሕመምዎ ምክንያት **ቤተሰብዎ** ምን ያህል ተቸግሯል? |
| How much of a problem did you have in doing things by yourself for relaxation or pleasure? | - This item was difficult to understand, and less applicable - Some simply understood it as ability to relax oneself or help oneself to get pleasure - It was even difficult for respondents to understand the meaning of relaxation and pleasure since these things are not common for people in rural areas of Ethiopia | - We did a lot of work to improve the translation and the adaptation - But, we could not improve the item since the concept is not applicable and relevant to this rural African context - So, this was one of the problematic items in the scale | **የሚያዝናናዎትን ወይም የሚያስደስትዎትን ነገር** ያለ ሌላ ሰው ድጋፍ ለማድረግ ምን ያህል ይከብድዎታል? |
